# Efficacy of Anti‐Adhesive Barriers on Outcomes Following Pediatric Abdominal Surgery: A Systematic Review and Meta‐Analysis

**DOI:** 10.1002/hsr2.71892

**Published:** 2026-02-22

**Authors:** Khang Duy Ricky Le, Shasha Haycock, Annie Jiao Wang

**Affiliations:** ^1^ Department of Surgery Northeast Health Wangaratta Wangaratta Victoria Australia; ^2^ Department of General Surgical Specialties The Royal Melbourne Hospital Melbourne Victoria Australia; ^3^ Geelong Clinical School Deakin University Geelong Victoria Australia; ^4^ Faculty of Medicine, Dentistry & Health Sciences The University of Melbourne Melbourne Victoria Australia

**Keywords:** abdominopelvic surgery, adhesions, anti‐adhesive barriers, bowel obstruction, laparoscopy, pediatric surgery, seprafilm

## Abstract

**Background and Aims:**

The efficacy of anti‐adhesive barriers in preventing clinically significant adhesional small bowel obstruction (ASBO) in the pediatric population is poorly defined. This review seeks to evaluate the utility of anti‐adhesive barriers in pediatric populations undergoing abdominopelvic surgery.

**Methods:**

A literature search was performed on Embase, Medline, Cochrane Centra,l and the World Health Organization (WHO) International Clinical Trials Registry Platform (ICTRP) on 20 August 2024. Our main outcome measures included ASBO, total complications, return to theater and surgical site infection.

**Results:**

A total of three studies were included in this review; however, two studies were published from the same group and institution. Separate analyses were performed due to the potential for overlapping cohorts. Analysis of two studies involving 912 patients comparing anti‐adhesive barriers (*n* = 462) to control (*n* = 440) suggested that anti‐adhesive barriers reduced the risk of ASBO compared with control (OR 0.39, 95% CI 0.19–0.84, *p* = 0.02). There is insufficient evidence to suggest that anti‐adhesive barriers were superior to control in reducing overall postoperative complications (OR 0.81, 95% CI 0.41–1.58, *p* = 0.63), surgical site infection (OR 0.95, 95% CI 0.35–2.57, *p* = 0.09).

**Conclusion:**

Anti‐adhesive barriers may have a role in the prevention of ASBO in pediatric populations undergoing open abdominopelvic surgery. However, this occurs on the background of moderate to high risk of bias, small sample size, and high clinical heterogeneity with poor control for confounders. Further prospective research is required to further validate these effects.

## 1 Introduction

Adhesions occur in up to 93% of patients following laparotomy [1]. While this forms a crucial component of healing for bowel anastomosis and abdominal closure, adhesions may also result in postoperative complications. These include abdominal pain and discomfort, anorexia, and malabsorption in addition to adhesional small bowel obstruction (ASBO), which may be further complicated by bowel ischemia and perforation [2].

As for the adult population, the highest readmission rate for pediatric adhesion‐related complications is within the first year of index operation [2, 3]. However, unlike in adults, where the majority of the ASBO resolve spontaneously with conservative management, failure rates for conservative management in children range from 45% to 100% [4]. Factors associated with the need for operative intervention include younger age, Caucasian background, and management in a pediatric center [4]. The type of adhesions also vary based on underlying pathology, with single bands associated with operations for simple mechanical obstruction, such as malrotation, while dense adhesions are associated with conditions involving inflammatory processes such as necrotizing enterocolitis [2]. Recurrent adhesive issues following surgical anti‐adhesion treatments remain a challenge in surgery. There is a relatively high recurrence rate following operated ASBO of 15.9% cumulative recurrence rate, with 5.8% requiring repeat surgical intervention [5]. Risk factors for recurrence reported include age below 40 years, diffuse or densely matted adhesions, and post‐operative complications, including surgical site infections [5].

Research into complications following laparotomy for neonates and children is limited by follow‐up, with the majority extending less than 5 years [4]. From long‐term studies into adults, it is known that problems may occur over 30 years after index operation [6]. This would place children, who have longer predicted lifespans, theoretically at greater risk of cumulative and long‐term complications. Due to the morbidity of adhesion‐related complications, the focus has shifted to the use of anti‐adhesive barriers to prevent clinically significant ASBO following gastrointestinal procedures [7]. Anti‐adhesive barriers are available as membranes, gels, and solutions. Commercially available options include Seprafim^R^, a hyaluronic acid/carboxymethyl cellulose membrane, Guardix^R^, a hyaluronic acid/carboxymethyl cellulose gel, and Interceed^R^, an oxidized regenerated cellulose liquid barrier [8]. Modified sodium hyaluronate (HA) and carboxymethylcellulose (CMC) anti‐adhesive barriers have been demonstrated to reduce the rate of adhesions in the adult population [7]. While no human studies are available that directly compare outcomes of the different anti‐adhesive barrier types, animal studies suggest that solid anti‐adhesive barrier products may be more apt at remaining in place during prolonged healing and thus more effective than gel products in adhesion prevention in a severe adhesion model. These studies also demonstrate that liquid anti‐adhesive barriers may over come the slipping effect due to the larger volume of liquid barrier, giving an equivocal outcome to solid barriers [8]. Despite evidence for reduced rate of adhesions, disadvantages and barriers to use of these barriers include perceptions that adhesions are not of clinical interest, lack of clarity around the specific anti‐adhesive indications, and cost considerations [9]. Further, there are specific concerns regarding anastomotic leaks and intra‐abdominal abscess with Seprafilm and anastomotic leaks, postoperative ileus, and wound complications with liquid hyaluronate‐based gels [10, 11]. The effect of anti‐adhesive barrier use on the rate of adhesions has yet to be characterized for pediatric populations. In this systematic review and meta‐analysis, we examine the efficacy of anti‐adhesive barriers that are used in cases of pediatric intraabdominal and pelvic surgery.

## 2 Methods

### 2.1 Review Protocol and Registration

This systematic review was performed with adherence to the Preferred Reporting Items for Systematic Reviews and Meta‐Analyses (PRISMA) guidelines [12]. The protocol was prospectively registered on the PROSPERO database (ID: CRD42024569337).

### 2.2 Literature Search

A literature search was performed on Embase, Medline, Cochrane Central, and the World Health Organization (WHO) International Clinical Trials Registry Platform (ICTRP) on 20 August 2024. The search strategy combines keywords, phrases, and medical subject headings (MeSH) related to anti‐adhesion barriers (such as Seprafilm) and pediatric populations. Hand‐searching was utilized to capture additional articles from relevant papers identified from the initial search. The search strategy is available as Supporting Information.

### 2.3 Inclusion and Exclusion Criteria

Peer‐reviewed full‐text articles available in the English language evaluating anti‐adhesive barriers in pediatric patients who underwent abdominopelvic surgery were considered for inclusion in this review. The inclusion criteria included articles that were randomized‐controlled trials, prospective cohort or case‐control studies, and retrospective cohort or case‐control studies. Additional inclusion criteria included articles that evaluated pediatric populations (age < 18 years) who underwent abdominal or pelvic surgery with application of an anti‐adhesive barrier. Exclusion criteria included studies that were of the following study designs: reviews, meta‐analyses, conference papers, posters, non‐human trials, letters, opinion articles, editorials, abstracts, commentaries, case reports, and case series. Additional exclusion criteria included articles that (1) evaluated adult populations (age ≥ 18 years) (2), utilized anti‐adhesive barriers for indications that did not require abdominal or pelvic surgery (3), had incomplete data, and (4) did not evaluate the outcomes of interest.

### 2.4 Literature Screening

Initial screening by title and abstract was performed by two independent investigators (KL, AW) for consideration of progression to full‐text analysis. Furthermore, articles where there was insufficient information also progressed to full‐text analysis. Full‐text analysis was performed independently by the same two investigators (KL, AW) with reference to inclusion and exclusion criteria. Disagreement at any stage was resolved by consensus. If consensus was not achieved, a third independent investigator (SH) was consulted for the final decision.

### 2.5 Outcomes

Outcomes of interest were related to the efficacy and safety of anti‐adhesive barriers when used in abdominopelvic surgery. These outcomes include acute ASBO, anastomotic leak, surgical site infection, ileus, intra‐abdominal abscess, enterocutaneous fistula, grade of adhesions, infection, severe complications (Clavien‐Dindo scores ≥ 3), length of stay, and total post‐operative complications. Grades of adhesions were defined as grade 0 (no adhesions), grade 1 (avascular, film‐like adhesions), grade 2 (limited vascularity, moderate thickness adhesions), and grade 3 (well vascularised, thick adhesions).

### 2.6 Data Extraction

Eligible studies were extracted for identifiers including author, year of publication, country of publication, and study design. Furthermore, patient characteristics and clinical indicators were extracted, which included age, sex, number of study participants, number of individuals who received an anti‐adhesive barrier compared to placebo, position of anti‐adhesive barrier placement, indication for surgery, surgical approach (such as laparoscopic, robotic, or laparotomy), and the outcomes of interest.

### 2.7 Data Synthesis and Statistical Analysis

Statistical analysis was performed utilizing Review Manager 5.4 (RevMan 5.4) software (Cochrane, London, United Kingdom). Homogeneous quantitative data were pooled where possible to allow meta‐analysis. A fixed‐effects model was employed if there was no clinical or statistical heterogeneity identified. If heterogeneity was present, a random‐effects model was employed. Odds ratios (OR), 95% confidence intervals (95% CI), and *p*‐values were extracted from the included studies or calculated where required. *p*‐values < 0.05 were considered significant. If there was heterogeneous reporting of continuous variables, conversion to single measures of effect was performed using the Wan method [13].

Heterogeneity was assessed using the Higgins I^2^ test with the following interpretation applied: less than 25% considered of low heterogeneity, between 25% and 50% considered as moderate, and above 50% considered as high heterogeneity [14]. Data from meta‐analyses were reported with Forest plots where possible. If homogeneous data were not available, relevant outcomes were reported descriptively.

### 2.8 Risk of Bias Assessment

Assessment of methodological rigor was performed using the Risk of Bias in Non‐randomized Studies of Interventions (ROBINS‐I) tool [15]. Risk of bias was assessed by two independent investigators (K.L., A.W.). Disagreement during this process was resolved by consensus. If consensus was not achieved, a third independent investigator (S.H.) was consulted for the final decision.

### 2.9 Subgroup and Sensitivity Analysis

Subgroup analysis or meta‐regression was performed to investigate sources of heterogeneity where relevant. Planned subgroup analyses included analysis based on location of anti‐adhesive barrier placement, analysis based on age groups defined as young child (age < 5 years), child (age 5–12 years), adolescent (age 13–18 years), and based on operative approach.

### 2.10 Analysis of Certainty of Evidence

The Grading of Recommendations Assessment, Development and Evaluation (GRADE) framework was used to assess the certainty of evidence.

## 3 Results

### 3.1 Literature Search

**FIGURE 1:**
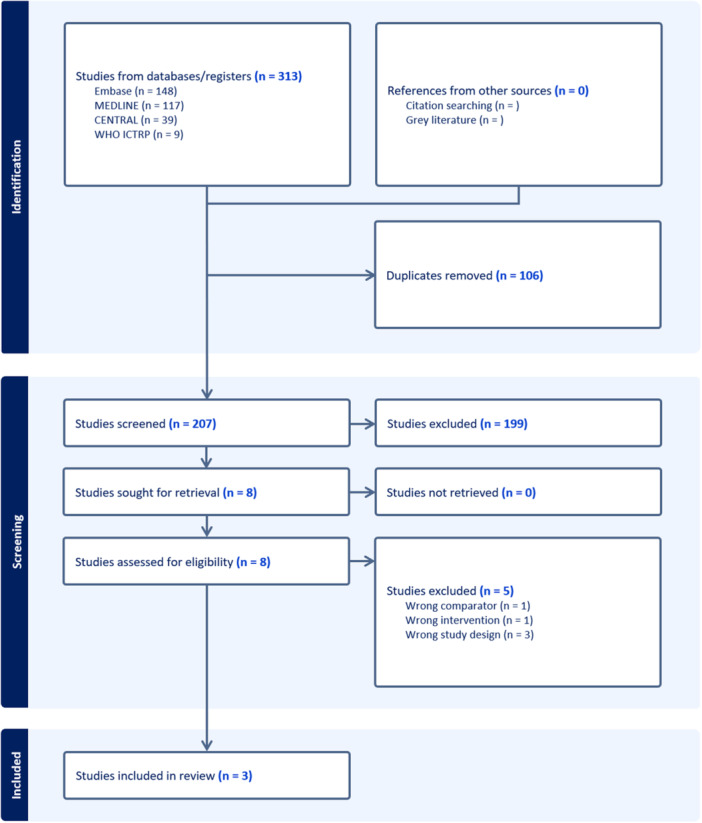
Flowchart demonstrating results of literature search.

Three hundred thirteen articles were retrieved from the literature search. Following the removal of duplicates, 207 articles were screened with 8 articles progressing to full‐text analysis. Three articles were subsequently included based on eligibility criteria. The PRISMA flowchart representing this process is shown in Figure 1.

### 3.2 Overview of Included Studies

Three studies comparing anti‐adhesive barriers compared to control in the pediatric population undergoing abdominal surgery were included in this review (Table 1) [16, 17, 18]. Two of these studies were published in Japan by the same group and institution. Two studies were randomized‐controlled trials, and one study was a cohort study that assessed an intervention arm that was prospectively followed and a control arm that was retrospectively examined. The remaining study was published in Ukraine. Given the potential for doubling of cohorts as well as limitations in the data reported, pooled demographic details of sample size, age, age category, and intervention were not performed. Anti‐adhesive barriers used included sodium hyaluronate gel (*n* = 1, 33%) and Seprafilm, a modified sodium hyaluronate and carboxymethylcellulose membrane (*n* = 2, 66%). Where gel was used, it was applied on the viscera prior to closure, whereas Seprafilm was placed per bowel prior to closure. Indications for surgery were highly heterogeneous and included common pediatric surgical conditions, such as acute appendicitis, to rare conditions such as biliary atresia. All cases were performed via an open approach, with no laparoscopic cases in any study.

**TABLE 1:** Overview of included studies.

Study	Study design	Country of study	Sample size (*n)*	Age (mean +/− SD)	Age category	Sex *(n)*	Barrier: Placebo *(n)*	Type of barrier	Location of barrier	Control	Surgical indication	Surgical approach
Kvashnina 2022 [16]	Prospective, randomized‐controlled patient blinded observational study	Ukraine	62	Control: 11.0+/−4.1 Intervention: 10.8+/−8.2	NR	M: 30 (control: 14, intervention: 16), F: 32 (control: 17, intervention: 15)	31, 31	Sodium hyaluronate gel	After abdominal lavage, 10 mg/ml SHG was applied on areas of visceral after abdo and parietal peritoneum before abdominal closure	Standard abdominal lavage before abdominal closure	Appendicitis	Laparotomy: 62 (midline laparotomy: 48; 77.4%)
Inoue 2005 [17]	Randomized controlled trial	Japan	122 161 (second analysis)	NR	Neonate (N = 56, C = 27, I = 29), Infant N = 48 (C = 22, I = 26), Child N = 18 (C = 6, I = 12)	M = 63 (Control 26; Intervention 37), F = 59 (Control 29; Intervention 30)	67, 55 89, 72 (second analysis)	Seprafilm (Genzyme Corporation, Cambridge, Mass)	One third of wound closure, appropriately sized Seprafilm sheet applied directly over bowel while lifting wound edge to maintain separation of tissue from abdominal wall, overlapping 3 cm beyond superior/inferior/lateral incision margins	Running absorbable braided suture for abdominal closure while keeping wound edge lifted	Duodenal atresia/stenosis, neuroblastoma, small intestinal atresia/stenosis, CDH, UC, Ovarian tumor, GI perforation, choledochcal cyst, intussusception, imperforate anus, biliary atresia, Hirschprungs, Gastroschisis/omphalocoele, hepatoblastoma, other	Upper abdominal transverse N = 76 (C: 31, I: 45), Lower abdominal transverse N = 32 (C: 18, I: 14), Midline N = 10 (C: 3, I: 7), Other N = 4 (C: 3, I: 1)
Inoue 2013 [18]	Prospective Intervention cohort compared with retrospective cohort control	Japan	850	NR	NR	M = 483 (I: 238, C: 245), F = 367 (I: 203, C: 164)	441, 409	Seprafilm (Genzyme Corporation, Cambridge, Mass)	Whole sheet of Seprafilm applied centered around incision at time of abdominal closure. For small incision, film sheet cut to approrpiate size ‐ whole pieces applied equally in all directions	Absorbable, synthetic, braided suture was used for peritoneal closure throughout the study period	Esophageal atresia, gastric perforation, gastric reflux, pyloric stnosis, small intestinal atresia/stenosis, intestinal perforation, intestinal malrotation, meconium ileus or peritonitis, adhesive bowel obstriction, Mekcel's diverticulum, acute appendicitis, intussisception, Hirshsprung's disease, imperforate anus, biliary atresia, congenital biliary dilatation, gastroschisis, omphalocele, congenital diaphragmmatic hernia, neuroblastoma, hepatoblastoma, nephroblastoma, sarcococcygeal/retroperitoneal teratoma, ovarian tumourl lymphangioa, other	Laparotomy: 850 (excluded laparoscopy)

Abbreviations: C, child; F, female; I, infant; M, male; N, neonate; NR, not reported; SD, standard deviation; SHG, sodium hyaluronate gel.

### 3.3 Adverse Events

#### 3.3.1 Total Complications

**FIGURE 2:**
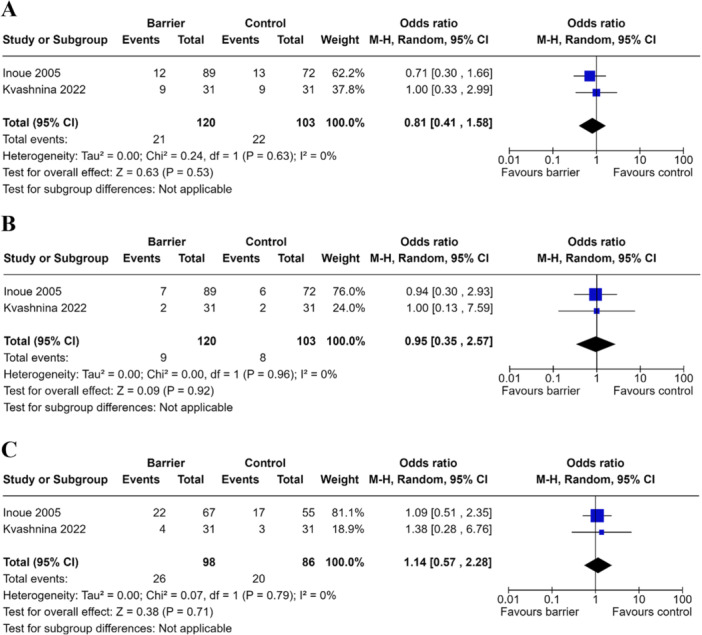
Meta‐analysis of anti‐adhesive barriers on post‐surgical complications. (A) Forest plot comparing total complication rates in pediatric population undergoing abdominal surgery receiving anti‐adhesive barrier or control. (B) Forest plot comparing surgical site infection rates in pediatric population undergoing abdominal surgery receiving anti‐adhesive barrier or control. (C) Forest plot comparing the risk of returning to theater for pediatric population undergoing abdominal surgery receiving anti‐adhesive barrier or control.

Two studies involving 223 patients comparing anti‐adhesive barrier (*n* = 120) to control (*n* = 103) were pooled for analysis [16, 17]. Meta‐analysis employing a random‐effects model demonstrated insufficient evidence to suggest anti‐adhesive barriers were superior to control in reducing overall post‐operative complications (OR 0.81, 95% CI 0.41–1.58, *p* = 0.63) (Figure 2A).

#### 3.3.2 Surgical Site Infection

Two studies involving 223 patients comparing anti‐adhesive barrier (*n* = 120) to control (*n* = 103) were pooled for analysis [16, 17]. Meta‐analysis employing a random‐effects model demonstrated insufficient evidence to suggest anti‐adhesive barriers were superior to control in reducing surgical site infection (OR 0.95, 95% CI 0.35–2.57, *p* = 0.09) (Figure 2B).

#### 3.3.3 Adhesive Small Bowel Obstruction

**FIGURE 3:**
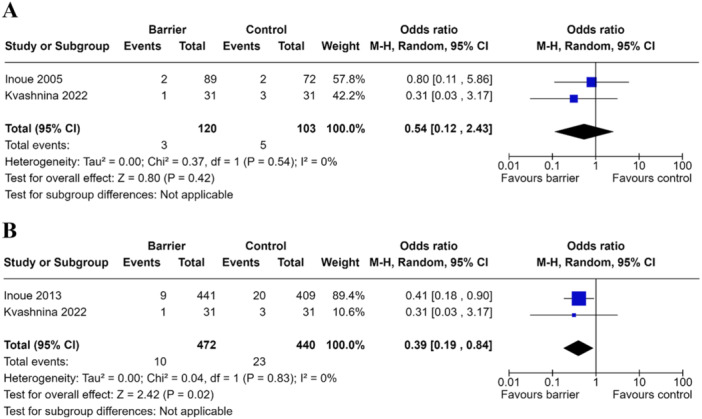
Meta‐analysis of anti‐adhesive barriers on adhesional small bowel obstruction outcomes. (A) Forest plot comparing adhesive small bowel obstruction rates in pediatric population undergoing abdominal surgery receiving anti‐adhesive barrier or control in Inoue 2005 (B). Forest plot comparing adhesive small bowel obstruction rates in pediatric population undergoing abdominal surgery receiving anti‐adhesive barrier or control in Inoue 2013.

Two separate analyses were performed due to overlapping cohorts. The first included two studies involving 223 patients comparing anti‐adhesive barrier (*n* = 120) to control (*n* = 103) [16, 17]. Meta‐analysis employing a random‐effects model demonstrated insufficient evidence to suggest anti‐adhesive barriers were superior to control in reducing ASBO (OR 0.54, 95% CI 0.12–2.43, *p* = 0.80) (Figure 3A). The second included two studies involving 912 patients comparing anti‐adhesive barrier (*n* = 462) to control (*n* = 440) (Figure 3B) [16, 18]. Meta‐analysis employing a random‐effects model demonstrated evidence to suggest anti‐adhesive barriers reduced the risk of ASBO compared to control (OR 0.39, 95% CI 0.19–0.84, *p* = 0.02) (Figure 3B).

#### 3.3.4 Return to Theater

Two studies involving 184 patients comparing anti‐adhesive barrier (*n* = 98) to control (*n* = 86) were pooled for analysis [16, 17]. Meta‐analysis employing a random‐effects model demonstrated insufficient evidence to suggest anti‐adhesive barriers were superior to control in reducing rates of return to theater (OR 1.14, 95% CI 0.57–2.28, *p* = 0.38) (Figure 2C).

#### 3.3.5 Other Outcomes

There was insufficient data for adequate meta‐analysis of the other *a priori* outcomes of interest. Despite this, individual studies reported on outcomes including grade of adhesions, anastomotic leak, and length of stay. Inoue et al. (2013) reported on grade of adhesions with Seprafilm compared to control. In their comparison, they measured adhesions by five grades as defined as grade 0 (no adhesions), grade 1 (film‐like adhesions that are not vascularised and easily blunt dissected), grade 2 (stronger adhesions that are beginning to vascularize but blunt dissection is still possible with the need for sharp dissection as necessary), grade 3 (strong adhesions with clear vascularization and need for sharp dissection), and grade 4 (very dense adhesions, adherent to organs and requiring sharp dissection) [18]. The authors demonstrated Seprafilm was associated with a greater amount of low‐grade adhesions from grade 0 to 2 compared to control (22/67; 32.84% compared to 13/55; 23.6%) [18]. Similarly, higher grade adhesions (grade 3 and 4) were less prevalent in the Seprafilm group compared to control (2/67; 2.99% vs. 4/55; 7.3%) [18]. Inoue et al. (2005) demonstrated with respect to anastomotic leak, comparable outcomes between Seprafilm (1/67) and control groups (1/55). Kvashnina et al. (2022) demonstrated similar length of stay between anti‐adhesive barrier group (16.4 days +/− 4.9) compared to control (15.7 days +/− 2.7) [17].

Additionally reported outcomes included length of intensive care unit (ICU) stay, time to bowels open, and anastomotic stricture. Kvashnina et al. (2022) demonstrated a similar length of ICU stay between the anti‐adhesive barrier group (5.8 days +/− 5.9) compared to control (5.1 days +/− 1.4) [16]. They also report similar time to bowels opening between the anti‐adhesive barrier group (2.9 days +/− 1.0) compared to control (3.3 days +/− 1.1) [16]. Lastly, Inoue et al. (2005) demonstrated comparable rates of anastomotic stricture rates in Seprafilm (1/67) compared to control (2/55) [17].

There was no data reported for ileus, intra‐abdominal abscess, enterocutaneous fistula, or severe complications.

### 3.4 Risk of Bias

**FIGURE 4:**
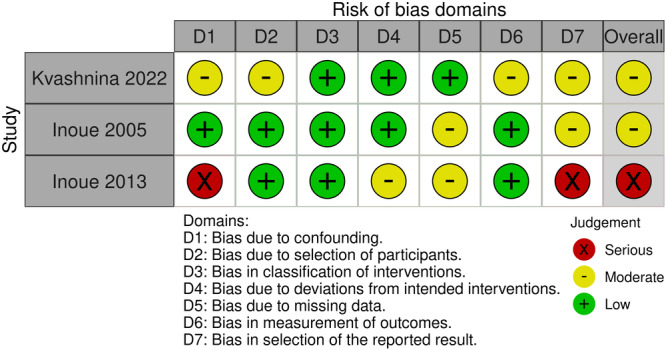
Risk of bias assessment using the ROBINS‐I tool.

Articles were deemed to be at moderate (*n* = 2) to serious (*n* = 1) risk of bias (Figure 4). Bias due to confounding variables included focus on laparotomies for appendicectomy (Kvashnina 2022) and comparisons of prospective populations with historical cohorts, which may differ in exposures such as surgical approaches (Inoue 2013) [16, 18]. Bias due to the selection of patients and missing data was due to a lack of concealment and a lack of reporting, respectively. Bias due to the measurement or reporting of outcomes was based on a lack of blinding.

### 3.5 Certainty of Evidence

There was an overall low to very low certainty of evidence attributed to imprecision, clinical heterogeneity, and risk of bias (Table 2).

**TABLE 2:** GRADE assessment of outcomes.

Outcome	Effect size (OR)	Certainty of evidence (GRADE)	Comments
Total complications	0.81 (2 studies)	⊕⊕◯◯ Low	Downgrade 1 point for imprecision and downgrade 1 point for inconsistency secondary to clinical heterogeneity. Publication bias unable to be assessed due to paucity in data.
Surgical site infection	0.95 (2 studies)	⊕⊕◯◯ Low	Downgrade 1 point for imprecision and downgrade 1 point for inconsistency secondary to clinical heterogeneity. Publication bias unable to be assessed due to paucity in data.
Adhesive small bowel obstruction (A)	0.54 (2 studies)	⊕⊕◯◯ Low	Downgrade 1 point for imprecision and downgrade 1 point for inconsistency secondary to clinical heterogeneity. Publication bias unable to be assessed due to paucity in data.
Adhesive small bowel obstruction (B)	0.39 (2 studies)	⊕◯◯◯ Very low	Downgrade 1 point for imprecision, downgrade 1 point for inconsistency secondary to clinical heterogeneity, and downgrade 1 point for risk of bias. Publication bias unable to be assessed due to paucity in data.
Return to theater	1.14 (2 studies)	⊕⊕◯◯ Low	Downgrade 1 point for imprecision and downgrade 1 point for inconsistency secondary to clinical heterogeneity. Publication bias unable to be assessed due to paucity in data.

Abbreviation: OR, odds ratio.

## 4 Discussion

This systematic review highlights a clear paucity of data relating to the use of anti‐adhesive barriers in the pediatric population. Meta‐analysis of the included studies demonstrates insufficient evidence to suggest superiority of anti‐adhesive barrier in reducing overall post‐operative complications, surgical site infections, or return to theater. Despite this, there was evidence to suggest anti‐adhesive barriers reduced the risk of ASBO in pediatric cohorts. This is an important outcome for pediatric patients post abdominal surgery due to greater likelihood of patients in this population requiring operative intervention for ASBO [4]. These findings, however, occur on the background of studies at moderate to serious risk of bias with significant clinical heterogeneity, and therefore, the certainty of this evidence is low.

Adhesions remain an inevitable consequence of intra‐abdominal and pelvic surgery. They confer the risk of significant complications, particularly for pediatric populations where higher rates of operative intervention occur due to ASBO. Traditionally, strategies to reduce the chance of adhesions include meticulous tissue handling and preference for more minimally invasive approaches to surgery [19]. Despite this, post‐operative adhesions and their downstream sequelae continue to challenge health systems. Anti‐adhesive barriers have been developed as a potential method to address the issue of adhesion formation. Early studies in the adult population have demonstrated that they can reduce the odds of clinically relevant ASBO [20]. More recently, Choy et al. in a systematic review and meta‐analysis of 17 adult studies, confirmed anti‐adhesive barriers reduced the odds of clinically significant ASBO by 55% without an increase in relevant complications, including anastomotic leak or infection [7]. This effect remains unexplored, however, in the pediatric population, and therefore, it is unclear whether these outcomes are translatable. Emerging pre‐clinical research in animal models has seen promising rates of adhesion reduction and prevention in animal models with the use of zwitterionic polymers, through application of a viscous injectable solution to the traumatized tissue surface. Post‐operative adhesions were completely and reliably prevented in > 93% of rat models [21]. Other promising research includes the use of human placental stem cells (hPSC) to effectively reduce abdominal adhesions by 65% compared with Seprafilm in rat models [22].

Despite our data identifying potential emerging evidence supporting the use of anti‐adhesive barriers in the setting of pediatric abdominopelvic surgery, it is important to note that the reduced incidence of ASBO did not correlate with reduced rates of return to theater. Thus, the use of anti‐adhesive barriers in children is likely preventing ASBO that resolve by conservative management, without change in the number of ASBO that require operative management. Therefore, unanswered questions remain surrounding the efficacy of these implants on clinically relevant ASBO. This contrasts with research into Seprafilm use in adults, where there was a small but significant reduction in the rate of reoperation for bowel obstruction following colorectal operations [23]. However, if reoperation is required for ASBO, these operations may be safer and more efficient in patients post‐Seprafilm due to the presence of milder adhesions and decreased adhesions at the wound site in the Seprafilm group [18]. This is supported by the significantly reduced operating time from starting the operation to starting the intestinal anastomosis for stoma closure in Seprafilm compared to control group and supports findings of previous adult studies [17, 24]. This is an important consideration for pediatric patients post‐abdominal surgery due to the higher associated reoperation rates for ASBO in this age group [4].

To the author's knowledge, this is the first systematic review and meta‐analysis on the topic of anti‐adhesive barriers in the pediatric population. However, there are important limitations to note within this study. The included articles were at moderate to serious risk of bias due to confounders, comparators using historical cohorts, missing data, and lack of appropriate blinding. Confounding factors, in particular, are likely to have a strong impact on results. The over‐representation of laparotomy for appendicectomy is noteworthy as the incidence of post‐appendicectomy adhesion‐related complications is low (0.35%–3%) compared to other intra‐abdominal procedures [4]. Furthermore, adhesion‐related complications are more prevalent in infants (4.7%) than older children (2.1%) [23]. Despite this, the youngest patient within one of the three studies was 3 years of age [16]. When considering comparators, Inoue et al. 2013 adopted the use of a historical control, which is another important confounder due to lack of control for the evolution of surgical techniques and equipment [18]. On examining individual studies, it is also important to note that two studies were from one institution with overlapping study bases [17, 18]. Given this, it was not possible to perform a meta‐analysis of all studies, as it was not possible to delineate cases that were duplicated in the analysis. This has implications for the generalizability of our results due to the small sample size and high heterogeneity. Additional sources of heterogeneity include the type of barrier used, ranging from films to gels, as well as how clinically relevant ASBO was defined. Moreover, given the paucity of evidence, no subgroup or sensitivity analyses were possible and it remains unclear the individual effects of these factors. Lastly, the cases included in the three studies include only pediatric laparotomy cases, which cannot be generalized to laparoscopic procedures. In the adult population, the use of anti‐adhesion cases has also been explored in laparoscopic operations [7]. Adoption in laparoscopic procedures in a pediatric population, however, is predicted to present additional challenges, such as the pediatric size ports for introducing the anti‐adhesive barrier. Taken together, the true impact of anti‐adhesive barrier usage may be misleadingly minimized in the present analysis. There is a need for larger, adequately powered prospective trials evaluating the effect of anti‐adhesive barriers to further understand the true impact of these implants on outcome for pediatric populations.

## 5 Conclusion

Our study highlights the general lack of data relating to the use of anti‐adhesive barriers in the pediatric population. Whilst our study suggests that there may be a role in the prevention of ABSO in pediatric populations undergoing open abdominopelvic surgery, which supports the current literature, there remains insufficient evidence to characterize its effect on postoperative and patient outcomes. Further study within this population is required to truly understand the efficacy and impact of these anti‐adhesive barriers long term.

## Author Contributions

**Khang Duy Ricky Le:** conceptualization, investigation, writing – original draft, writing – review and editing, validation, visualization, methodology, formal analysis, project administration, resources, data curation, supervision. **Shasha Haycock:** investigation, writing – original draft, writing – review and editing, validation, methodology, formal analysis, data curation, resources. **Annie Jiao Wang:** resources, data curation, formal analysis, writing – review and editing, writing – original draft, investigation, methodology, validation.

## Conflicts of Interest

The authors declare no conflicts of interest.

## Transparency Statement

The lead author, Khang Duy Ricky Le, affirms that this manuscript is an honest, accurate, and transparent account of the study being reported; that no important aspects of the study have been omitted; and that any discrepancies from the study as planned (and, if relevant, registered) have been explained.

## Supporting information

Supplementary material‐appendix.

Supplementary material‐PRISMA Checklist.

## Data Availability

Data sharing is not applicable to this article as no new data were created or analyzed in this study. All data references and utilized in the generation of this manuscript were obtained from peer‐reviewed published academic literature. The authors confirm that the data supporting the findings of this study are available within the article and its reference list.
