# Supplementary material for: Efficacy of Anti‐Adhesive Barriers on Outcomes Following Pediatric Abdominal Surgery: A Systematic Review and Meta‐Analysis
Source: Health Sci Rep. 2026 Feb 22;9(2):e71892. doi: 10.1002/hsr2.71892 (PMC12927938; doi:10.1002/hsr2.71892)
Supplement: Supplementary file 1 — Supplementary material‐appendix. [file HSR2-9-e71892-s002.docx]

**Appendix**

Appendix A: Search strategy

| 1 | (Seprafilm or Sepracoat or Hyalobarrier or Hyaloglide or Interceed or Parietex or Sprayshield or AdSpray).mp. |
| --- | --- |
| 2 | Hyaluronic Acid/ and (Membranes, Artificial/ or Tissue Adhesions/) |
| 3 | (Biocompatible Materials/ or gels/) and Tissue Adhesions/ |
| 4 | ((hyaluron* or hydrogel) adj4 (membrane* or barrier* or gel or agent* or material* or film*)).mp. |
| 5 | Tissue Adhesions/ or (adhesion* or antiadhesion*).mp. |
| 6 | 4 and 5 |
| 7 | ((adhesion or antiadhesion or Adhesion Prevent*) adj3 (barrier* or agent* or material* or film* or gel)).mp. |
| 8 | 1 or 2 or 3 or 6 or 7 |
| 9 | adolescent/ or exp child/ or exp infant/ |
| 10 | (child or children or p?ediatric or infant* or adolescen*).tw,kw. |
| 11 | 9 or 10 |
| 12 | 8 and 11 |
| 13 | 12 not (animal/ not human/) |
| 14 | limit 13 to english language |
